# HDL metabolism and functions impacting on cell cholesterol homeostasis are specifically altered in patients with abdominal aortic aneurysm

**DOI:** 10.3389/fimmu.2022.935241

**Published:** 2022-09-12

**Authors:** Maria Pia Adorni, Marcella Palumbo, Cinzia Marchi, Francesca Zimetti, Alice Ossoli, Marta Turri, Franco Bernini, Ivana Hollan, Jiří Moláček, Vladislav Treska, Nicoletta Ronda

**Affiliations:** ^1^ Department of Medicine and Surgery, Unit of Neuroscience, University of Parma, Via Volturno 39/F, Parma, Italy; ^2^ Department of Food and Drug, University of Parma, Parco Area delle Scienze 27/A, Parma, Italy; ^3^ Centro E. Grossi Paoletti, Dipartimento di Scienze Farmacologiche e Biomolecolari, Università degli Studi di Milano, Milano, Italy; ^4^ Lillehammer Hospital for Rheumatic Diseases, M. Grundtvigs veg 6, Lillehammer, Norway and Brigham and Women’s Hospital, Cardiology Division, Boston, United States; ^5^ Department of Vascular Surgery, Faculty of Medicine and University Hospital in Plzen, Charles University Ovocný trh 5 Prague 1, Plzen, Czechia

**Keywords:** vascular biology, arterial aneurysm, inflammation, lecithin cholesterol acyltransferase, cholesteryl ester transfer protein, cholesterol efflux, ABCG1, ABCA1

## Abstract

**Background:**

The etiopathogenesis of abdominal aortic aneurysm (AAA) is still unclarified, but vascular inflammation and matrix metalloproteases activation have a recognized role in AAA development and progression. Circulating lipoproteins are involved in tissue inflammation and repair, particularly through the regulation of intracellular cholesterol, whose excess is associated to cell damage and proinflammatory activation. We analyzed lipoprotein metabolism and function in AAA and in control vasculopathic patients, to highlight possible non-atherosclerosis-related, specific abnormalities.

**Methods:**

We measured fluorometrically serum esterified/total cholesterol ratio, as an index of lecithin-cholesterol acyltransferase (LCAT) activity, and cholesteryl ester transfer protein (CETP) activity in patients referred to vascular surgery either for AAA (n=30) or stenotic aortic/peripheral atherosclerosis (n=21) having similar burden of cardiovascular risk factors and disease. We measured high-density lipoprotein (HDL)-cholesterol efflux capacity (CEC), through the ATP-binding cassette G1 (ABCG1) and A1 (ABCA1) pathways and serum cell cholesterol loading capacity (CLC), by radioisotopic and fluorimetric methods, respectively.

**Results:**

We found higher LCAT (+23%; p < 0.0001) and CETP (+49%; p < 0.0001) activity in AAA sera. HDL ABCG1-CEC was lower (−16%; p < 0.001) and ABCA1-CEC was higher (+31.7%; p < 0.0001) in AAA. Stratification suggests that smoking may partly contribute to these modifications. CEC and CETP activity correlated with CLC only in AAA.

**Conclusions:**

We demonstrated that compared to patients with stenotic atherosclerosis, patients with AAA had altered HDL metabolism and functions involved in their anti-inflammatory and tissue repair activity, particularly through the ABCG1-related intracellular signaling. Clarifying the relevance of this mechanism for AAA evolution might help in developing new diagnostic parameters and therapeutic targets for the early management of this condition.

## Introduction

Abdominal aortic aneurysm (AAA) is a very common pathology of the aorta with a complex and not fully understood etiopathogenesis characterized by a loss of elastic lamina and smooth muscle cells, inflammation, and matrix metalloproteases activation (1).

Data on the relationship between aortic aneurysm and lipid metabolism are limited. Mendelian randomization studies indicate a relationship between AAA and increased serum levels of total and low-density lipoprotein (LDL)-cholesterol (2). Cholesterol homeostasis within the arterial wall in AAA seems also to be disturbed, as abnormal expression of genes coding for molecules involved in cell cholesterol handling has been described within the aortic aneurysms (3, 4).

Cell cholesterol homeostasis is largely influenced by circulating lipoprotein levels and function. LCAT and CETP are major players in serum lipoprotein metabolism and remodeling (5). Their activity modulates not only concentrations but also quality and functions of lipoproteins. Little is known on the possible role of LCAT and CETP activity or lipoprotein dysfunction in the development and progression of AAA. The functionality of serum lipoproteins is very important not only for their activity as lipid carriers but also as regulators of tissue inflammation and repair (6, 7). Indeed, inflammation is one of the main mechanisms of the arterial wall modifications leading to dilatation in AAA. Altered polarization, towards a proinflammatory phenotype, of arterial macrophages has been reported (8). HDLs, through their capacity to promote cell cholesterol efflux (CEC), oppose to foam cell formation by unloading macrophages from excess cholesterol *via* efflux mediated mainly by the ATP-binding cassette transporters A1 and G1 (ABCA1 and ABCG1) (9). In addition, the scavenger receptor class B type I (SR-BI) is involved in the efflux process by facilitating the aqueous diffusion of cholesterol out of cells following the cholesterol gradient between cells and HDL (10, 11), however exerting a negligible contribution to overall cholesterol efflux from macrophages, as previously demonstrated (12).

HDL CEC is also associated with intracellular signaling resulting in the inhibition of inflammatory and immune reactions (13), particularly through the interaction with the membrane cholesterol transporters ABCG1 and ABCA1. The capacity of HDL to interact with specific membrane cholesterol transporters depends on the maturation process that they undergo in the serum, which generates different HDL particles subclasses (14). Spherical and mature HDL particles, resulting from the activity of LCAT that esterifies free cholesterol in nascent HDL (15), promote cholesterol aqueous diffusion (16) and, most importantly, cholesterol efflux through ABCG1 (17). Instead, lipid poor preβ-HDL particles are produced not only by the liver but also by the activity of CETP, which removes LCAT-derived cholesterol ester on mature HDL (18). These particles promote cholesterol efflux mostly interacting specifically with ABCA1 (19, 20), although a previous study demonstrated that preβ-HDL particles larger than 7.8 nm can also favor ABCG1-mediated cell cholesterol efflux (21).

ABCG1 activity seems to be particularly important for the control of inflammation. For example, ABCG1 but not ABCA1 myeloid-specific knockdown is associated with inflammation and granuloma formation (22). ABCG1 activity also modulates macrophage and T-cell activation (23). We have also reported that only ABCG1- and not ABCA1-mediated CEC inversely correlated to disease activity in patients with rheumatoid arthritis (24).

A previous work reports impaired CEC, measured as macrophage cholesterol efflux with no distinction between single pathways, in patients with AAA and in controls differing in serum lipid profile (25). In a following work from the same group, comparing CEC in patients with various stages of the disease, the authors concluded that HDL CEC is not involved in AAA progression (26). Thus, the question of the role of HDL CEC in AAA is still open. Most importantly, no information is currently available with respect to specific ABCA1- and ABCG1-CEC or on HDL remodeling serum enzymes activity in AAA patients compared to a control population with the same serum lipid profile and cardiovascular disease burden.

Opposite to HDL, LDL promote cell cholesterol accumulation and inflammation, especially when chemically modified by oxidation (27). The importance of LDL-cholesterol serum levels for cardiovascular risk is unquestioned, but equal levels might associate with different pathogenic potential due to qualitative and functional differences (28, 29). In patients with AAA, no data are available on serum cholesterol loading capacity (CLC), a measure of the overall serum *in vitro* ability to increase intracellular cholesterol content (30).

As intracellular cholesterol homeostasis in macrophages and other vascular cells is associated with proinflammatory activation within tissues, also independently from systemic inflammation (31), in this study, we looked for abnormalities of serum lipoprotein metabolism/functions impacting on cell cholesterol content, which could be specific for patients with AAA. To this aim, we tested the serum from a group of patients with AAA and from a matched control group of patients presenting the same lipid profile, cardiovascular comorbidities, and statin use, differing from AAA patients for the absence of aortic dilatation and for the presence of stenotic vascular disease. We analyzed serum esterified/total cholesterol ratio as an index of LCAT activity and directly measured CETP activity. Then, we measured serum CEC through specific pathways of cholesterol efflux, mediated by ABCG1, ABCA1, and aqueous diffusion. We also measured serum CLC and looked for correlations between CEC and CLC with other parameters. Finally, since strong risk factors for AAA is cigarette smoking (32), we evaluated the effect of smoking habit on the above parameters.

## Material and methods

### Study participants

All participants gave informed consent before inclusion in the study, which conformed to the principles outlined in the Declaration of Helsinki, and was approved by the Ethical Committee of the Faculty of Medicine in Plzen (12/04/2014).

Among the patients referred to vascular surgery (University Hospital, Plzen), we consecutively selected patients with abdominal aortic aneurysm undergoing open resection or endovascular procedure-EVAR (AAA, n=30) and control patients with atherosclerotic stenosis in the abdominal aorta or other arteries supplying lower extremities, undergoing aortobifemoral bypass surgery (non-AAA CTRL, n=21). In all cases, CT angiographic study had been performed. Sample size was calculated *a priori* using the G-Power Software. Blood drawing was performed after overnight fasting. Serum aliquots were stored at −80°C.

Concentrations of triglycerides, total cholesterol, and HDL-cholesterol were measured using enzymatic methods with photometric detection on Cobas system (Cobas 8000 Analyzer, Cobas c702 module, Roche Diagnostics). LDL-cholesterol was calculated using the Friedwald equation. C-reactive protein (CRP) was measured with a commercially available high-sensitivity ELISA kit (Thermo Fisher Scientifics, Italy).

### LCAT activity index

We evaluated the serum esterified/total cholesterol ratio as an index of LCAT activity (33), in triplicate. Sera were analyzed fluorometrically for total and free cholesterol, in the presence and absence of the cholesterol esters-hydrolyzing enzyme cholesterol esterase, respectively, by using the Amplex Red Cholesterol Assay Kit (Molecular Probes). Esterified cholesterol was then calculated.

### CETP activity

Serum CETP activity was measured fluorometrically, in triplicate, using the commercially available CETP Activity Assay Kit (Sigma Aldrich), as previously described (34).

### HDL-cholesterol efflux capacity

CEC mediated by specific pathways of cholesterol efflux was studied as previously described (24), in triplicate. Briefly, for ABCG1-CEC, Chinese hamster ovary cells transfected and not transfected with the human ABCG1 gene were used. For AD and ABCA1-CEC, we used J774 murine macrophages, in basal conditions or incubated with a cAMP analogue (cpt-cAMP, 0.3 mM; Sigma-Aldrich, Milano, Italy) inducing ABCA1 expression. In all assays, cells were labeled with [1,2-^3^H]-cholesterol (PerkinElmer, Milano, Italy) for 24 h and treated for 4 or 6 h with 1%–2% (v/v) apoB-depleted serum, obtained by PEG precipitation (24). This procedure, which provides biological samples with only HDLs, is comparable to HDL isolation through ultracentrifugation for the CEC study (35). HDL-CEC was expressed as percentage of the radioactivity released into the medium over the total radioactivity incorporated by cells.

### Serum HDL preβ-migrating particles

Serum preβ-HDL content was assessed in 10 AAA patients and in 10 control patients after separation by 2D electrophoresis. Agarose gel electrophoresis was followed by non-denaturing gradient gel electrophoresis and then followed by immunodetection against human apoA-I. Serum preβ-HDL content was expressed as a percentage of total apoA-I signal (36).

### Serum cholesterol loading capacity

THP-1-derived macrophages were incubated with 5% lipoprotein deficient serum (LPDS, Sigma-Aldrich) for 24 h and subsequently exposed to 10% (v/v) whole serum for 24 h, in triplicate. Cells were lysed in 1% sodium cholate solution (Sigma-Aldrich) with 10 U/ml DNase (Sigma-Aldrich). Cholesterol was measured fluorometrically using the Amplex Red Cholesterol Assay Kit (Molecular Probes, Eugene, OR) (37). CLC was expressed as micrograms cholesterol/milligram protein.

### Statistical analyses

Statistical analyses were performed using Prism (version 7.0) (GraphPad Inc., San Diego, CA). Data were expressed as mean ± SD. A comparison between two groups was performed using the unpaired two-tailed Student’s t-test or Mann–Whitney test for parameters with normal and skewed distribution, respectively. Categorical variables were compared with the Chi-square test. The relationship between parameters were evaluated by linear regression analysis or correlation, as specified. Significant differences were defined by p<0.05.

## Results

### Patient characteristics

Serum lipid profile and the other clinical parameters including CRP did not differ between AAA and control patients, except for age, in which it was slightly higher in the AAA group (**Table 1**). Details on serum lipid profile are shown in **Supplementary Figure 1S**.

**Table 1:** Clinical and biochemical parameters of control and AAA patients.

	Control patients	AAA patients	
**Gender**	71% (15/21) male	77% (23/30) male	NS
**Age** (years)	62 ± 10.14	68 ± 6.30	p<0.05
**BMI**	27.17 ± 3.81	26.85 ± 4.38	NS
**Smoke**	76% (16/21) yes	77% (23/30) yes	NS
**Previous CVE**	48% (10/21) yes	37% (11/30) yes	NS
**CIHD**	38% (8/21) yes	30% (9/30) yes	NS
**LEAD**	43% (9/21) yes	30% (9/30) yes	NS
**Hypertension**	67% (14/21) yes	77% (23/30) yes	NS
**DM**	24% (5/21) yes	27% (8/30) yes	NS
**TC** (mM/L)	4.33 ± 1.43	4.51 ± 1.22	NS
**LDL-C** (mM/L)	2.18 ± 0.93	2.12 ± 0.89	NS
**HDL-C** (mM/L)	1.78 ± 0.72	1.71 ± 0.99	NS
**Non HDL-C** (mM/L)	2.60 ± 1.22	2.83 ± 1.17	NS
**TG** (mM/L)	1.40 ± 0.69	1.88 ± 1.22	NS
**Statin use**	19% (4/21) yes	30% (9/30) yes	NS
**CRP** (mg/L)	2.19 ± 0.57	2.09 ± 0.76	NS

Values express mean ± SD or % and number of patients with the described variable in each group.

BMI, body mass index; CVE, cardiovascular events; CIHD, chronic ischemic heart disease; LEAD, lower extremity arterial disease; DM, diabetes mellitus; TC, total serum cholesterol; LDL-C, serum low-density lipoprotein cholesterol; HDL-C, serum high-density lipoprotein cholesterol; TG, serum triglycerides; CRP, serum C-reactive protein. NS, not significant.

### LCAT activity

**Figure 1:**
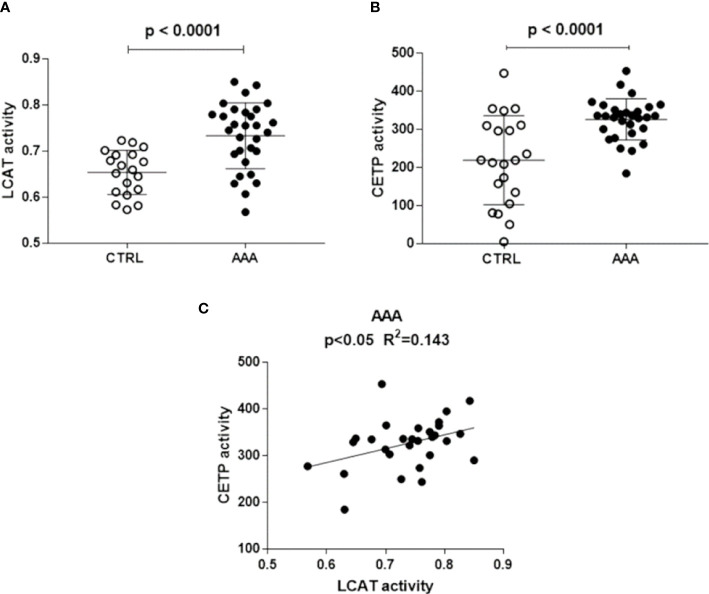
LCAT and CETP activity in AAA and non AAA control patients. LCAT and CETP activity is shown in panels **(A, B)**. Mean and SD for each group are reported. Unpaired two-tailed Student’s t-test for parameters with normal distribution was used. The significant relationship between LCAT and CETP activity, found only in AAA patients by linear regression analysis, is shown in panel **(C)**.

LCAT activity, measured indirectly through the serum esterified/total cholesterol ratio, was significantly higher in AAA than in control patients (+ 23%, p<0.0001) (**Figure 1A**).

### CETP activity

CETP activity was significantly higher in AAA than in control patients (+49%, p<0.0001) (**Figure 1B**). A direct significant relationship between LCAT and CETP activity (R^2^ = 0.143, p<0.05) was found only in the AAA group (**Figure 1C**).

### HDL cholesterol efflux capacity

**Figure 2:**
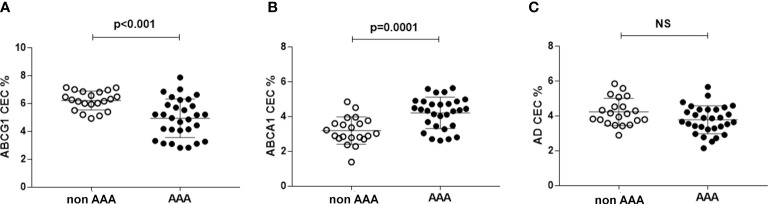
HDL CEC in AAA and non-AAA control patients. Panel **(A)** refers to ABCG1-mediated CEC; panel **(B)** refers to ABCA1-mediated CEC; panel **(C)** referes to AD-mediated CEC. The mean and SD for each group are reported. Unpaired two-tailed Student’s t-test for parameters with normal distribution was used. NS, not significant.

ABCG1-CEC in AAA was 16% lower than in control patients (p<0.001) and with more dispersed values (**Figure 2A**). On the contrary, ABCA1-CEC was significantly higher in patients with AAA (+31.7%, p<0.0001) (**Figure 2B**). Aqueous diffusion-CEC showed a non-statistically significant trend towards a reduction in AAA (**Figure 2C**).

### Correlation of HDL-CEC with other parameters

**Figure 3:**
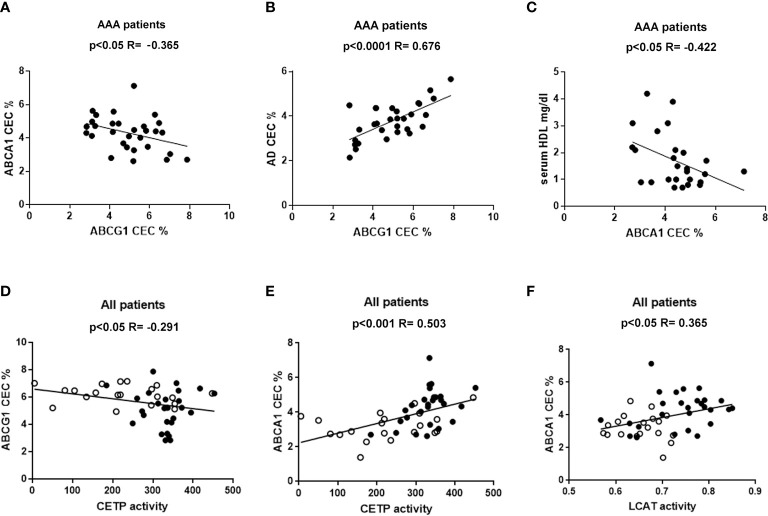
Correlations between HDL CEC and other parameters. Panel **(A–C)** refer to statistically significant correlations found only in AAA patients. Panels **(D–F)** show the statistically significant correlation found considering together non-AAA control (ο) and AAA patients (●).

None of the efflux pathways correlated with other parameters in the control group of patients (data not shown). Conversely, in the AAA group, ABCG1-CEC correlated inversely with ABCA1-CEC (R = −0.365, p<0.05) (**Figure 3A**) and directly with aqueous diffusion-CEC (R = 0.676, p<0.0001) (**Figure 3B**). Moreover, we found an inverse correlation between ABCA1-CEC and serum HDL levels in the same group (R = −0.422; p<0.05) (**Figure 3C**).

Considering all patients together, ABCG1-CEC and CETP activity were inversely correlated (R= −0.291, p<0.05) (**Figure 3D**), while ABCA1-CEC was directly correlated both with LCAT and CETP activity (R = 0.503, p<0.001, and R= 0.365, p<0.05, respectively) (**Figures 3E, F).** ABCA1-CEC was significantly directly correlated with CETP, also considering AAA patients separately (R=0.434, p<0.05).

### Effect of smoking habit on HDL-CEC and activity of LCAT and CETP

**Figure 4:**
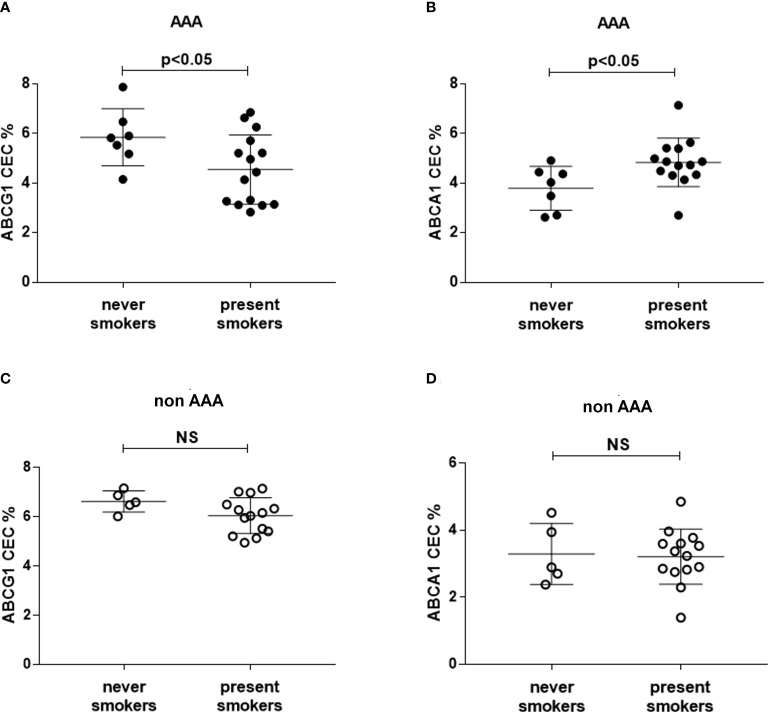
HDL CEC in smoker and non-smoker patients. ABCG1-CEC **(A, C)** and ABCA1-CEC **(B, D)** values were stratified into never (N=5 and N=7 for controls and AAA, respectively) and present smokers (N=14 and N=15 for controls and AAA, respectively). Mean and SD for each group are reported. The unpaired Mann–Whitney test for statistical analysis of parameters with skewed distribution was applied. NS, not significant.

We selected patients who were smokers or non-smokers at the time of the study, excluding past smokers, in the two groups. In AAA, ABCG1-CEC was significantly lower and ABCA1-CEC was significantly higher in smokers than in non-smokers (−22.2%, p<0.05, +27.5, p<0.05, respectively) (**Figures 4A, B**), with no difference in serum lipid profile (data not shown). We found no difference between smokers and non-smokers within the control patient group (**Figures 4C, D**).

Stratification by smoking did not produce significant differences in LCAT and CETP activity (data not shown).

### Serum HDL preβ-migrating particles

**Figure 5:**
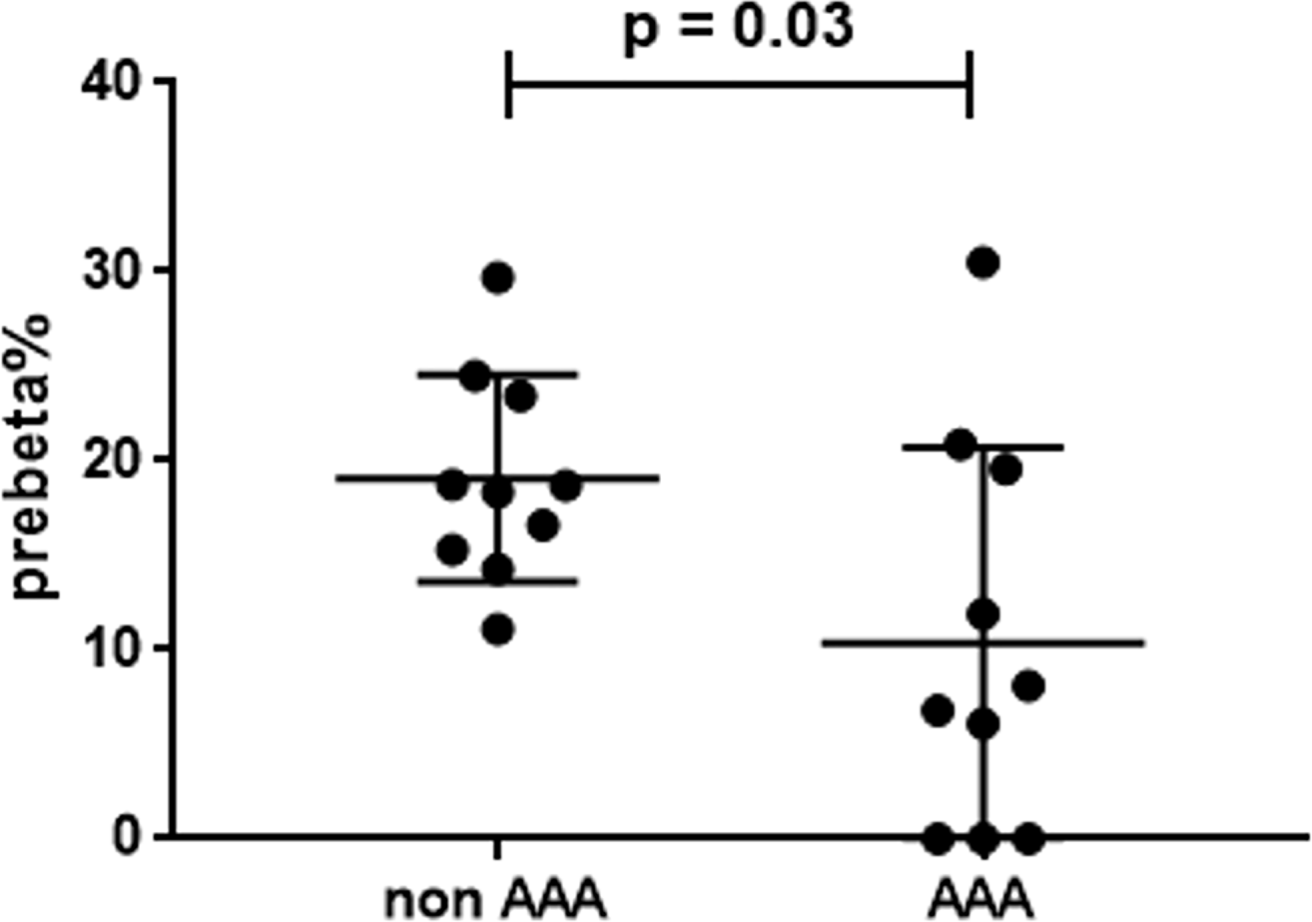
Serum preβ-HDL particles in AAA and non-AAA control patients. The mean and SD for each group are reported. The unpaired two-tailed Student’s t-test was applied.

The measurement of serum preβ particles as percentage over total apoAI signal showed significantly lower levels in AAA patients (19.1 ± 5.5%) than in control patients (10.4 ± 10.3%) (**Figure 5**).

### Serum cholesterol loading capacity

**Figure 6:**
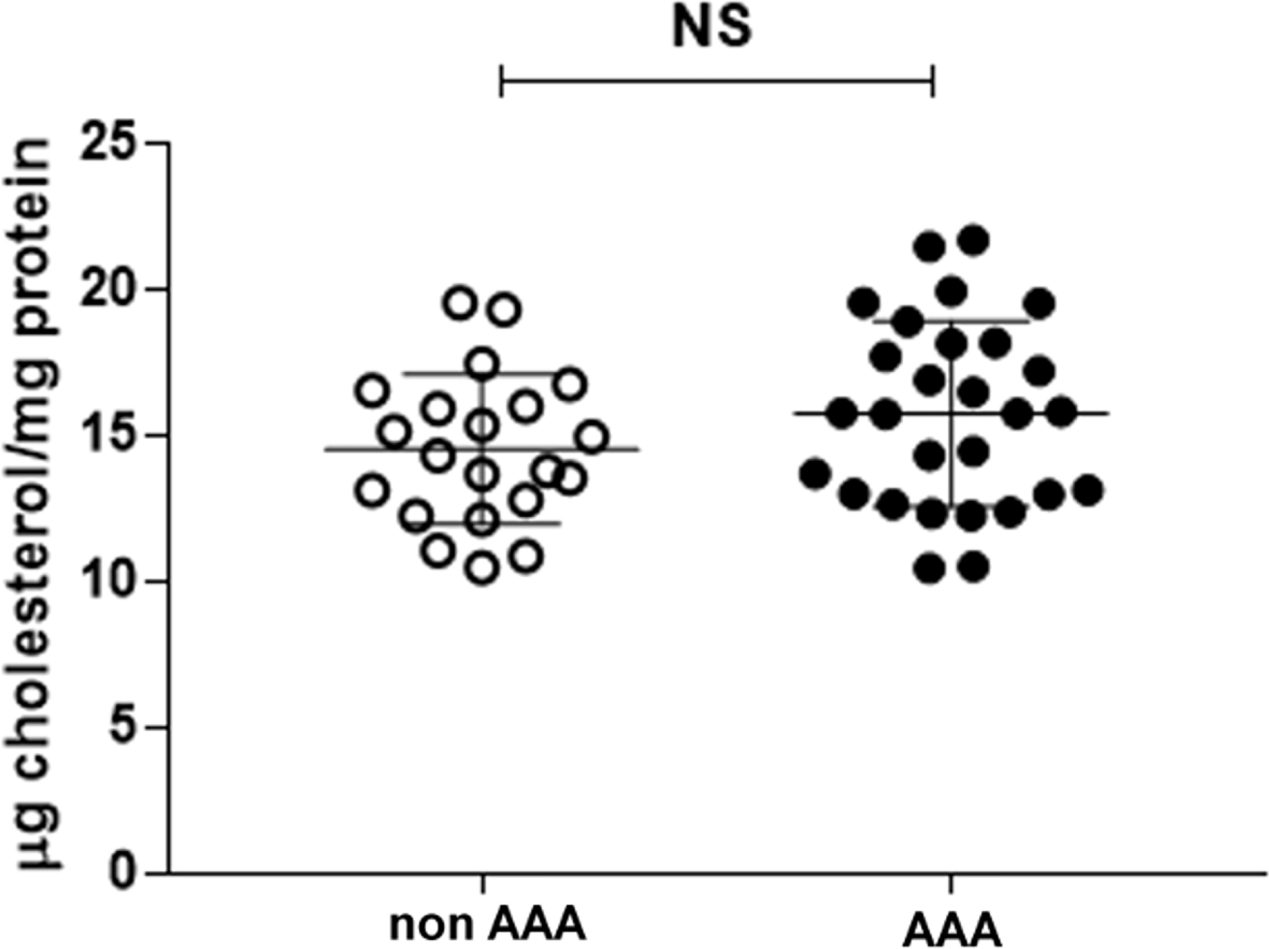
Serum CLC in AAA and non-AAA control patients. The mean and SD for each group are reported. The unpaired two-tailed Student’s t-test was applied. NS, not significant.

CLC was not different between the two groups of patients (**Figure 6**) or between smokers and non-smokers within each group (data not shown).

### Correlation of serum CLC with other parameters

**Figure 7:**
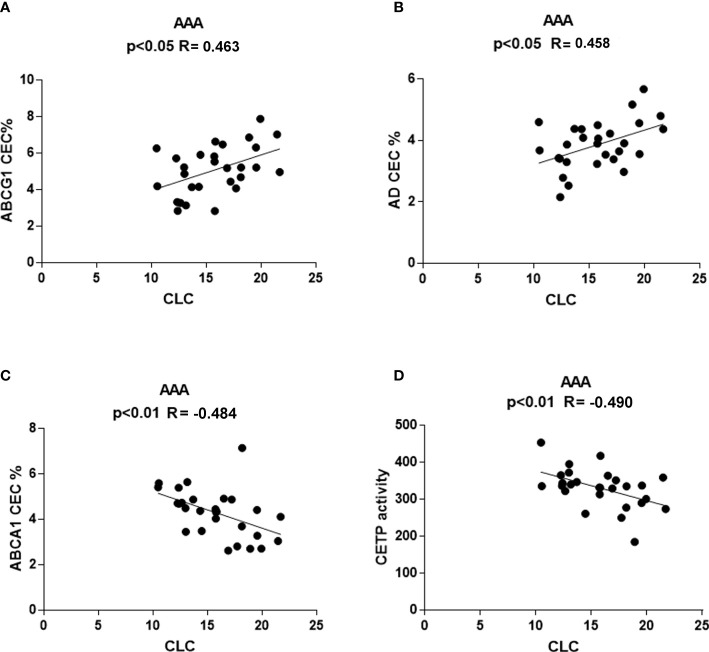
Correlations between serum CLC and other parameters in AAA patients. Positive correlation of CLC with ABCG1-CEC **(A)** and AD-CEC **(B)** and inverse correlation of CLC with ABCA1-CEC and CETP **(C)** activity **(D)** were found only in AAA patients.

No correlation was found between CLC and other parameters in the control group (data not shown). Conversely, in the AAA group, CLC correlated directly with ABCG1-CEC (R = 0.463, p<0.05) and with aqueous diffusion-CEC (R = 0.458, p<0.05) (**Figures 7A, B**). An inverse correlation was found between CLC and ABCA1-CEC (R = −0.484, p<0.01), and with CETP activity (R= −0.490, p<0.01) (**Figures 7C, D**).

## Discussion

Circulating lipoproteins are involved in tissue inflammation and repair (38, 39), events that are pivotal for AAA formation and progression (1), particularly through the regulation of intracellular cholesterol. Indeed, cell cholesterol homeostasis is involved in cell damage and proinflammatory activation (13, 40) and in AAA development (41). In this work, we evaluated lipoprotein metabolism and functions in AAA patients compared to a group of patients presenting no aortic dilatation but rather arterial stenosis, similar lipid profile, and the same main cardiovascular risk factors and prevalence of cardiovascular disease. We studied HDL remodeling and functions related to cell cholesterol homeostasis because cell cholesterol balance in macrophages, endothelial cells, and smooth muscle cells has a significant impact on inflammation and tissue repair, potentially very important in AAA (13, 42–44).

The augmented LCAT and CETP activity that we found in AAA patients is possibly related to the genetic background (45) of AAA. The direct correlation between the two enzymes activity, detected only in the AAA group, led us to hypothesize that, in these patients, increased LCAT provides CETP with abundant substrate, i.e., HDL esterified cholesterol, leading to accelerated HDL remodeling (20, 46). However, it cannot be excluded that also such association derives from a genetic or epigenetic, metabolic milieu impacting on the expression of both enzymes in AAA patients (41). Our data on LCAT and CETP activity differ from those presented in a previous report showing no difference between AAA patients and controls (25), possibly due to differences in subject cohorts.

HDL metabolism is associated with the formation of subsets of HDL particles differing for dimension and composition in terms of lipids and other carried molecules. The measurement of HDL-CEC specific for single cholesterol transporters provides information on the function and distribution of the various HDL subpopulations. For example, the significantly lower ABCG1-CEC and higher ABCA1-CEC found in AAA patients, both independently of HDL concentration (equal in the two groups of patients), suggests a reduction or a dysfunction of mature particles, specific for ABCG1 (47). This is in line with our hypothesis of an accelerated HDL turnover. Indeed, the inverse correlation between ABCG1-CEC and ABCA1-CEC found only in the AAA group is consistent with the described mechanisms of HDL remodeling. The actual role of serum enzyme activity in this process is confirmed by the inverse and direct correlation of CETP activity with ABCG1-CEC and ABCA1-CEC, respectively. ABCG1-mediated efflux mainly occurs to mature HDL particles, which represent the buoyant plasma HDL, but it can also be promoted by the discoidal preβ-migrating HDL with a size of ≥7.8 nm (21). However, mature HDL are more efficient in accepting cholesterol from the ABCG1 transporter and are much more abundant in serum as compared to preβ-HDL particles (20, 47). Thus, the ABCG1 CEC variations are likely related to the mature HDL particles reduction and or dysfunction. On the other hand, we also observed lower serum preβ particles in AAA compared to control patients, possibly due to HDL increased lipidation by LCAT. The higher ABCA1-CEC that we found in AAA with respect to control patients may be due to the activity of free apoAI as cholesterol acceptor.

Although the final intracellular cholesterol content might be unaffected by the CEC alterations that we found in AAA patients, ABCG1-CEC reduction could be relevant to aneurysm development or expansion. In fact, the cholesterol efflux through the ABCG1 transporter is coupled to intracellular anti-inflammatory signaling in macrophages and endothelial cells, and clinical studies demonstrated specific association between ABCG1-CEC impairment and inflammation indexes (24, 48, 49). Inflammation is considered a very important factor in vessel wall abnormalities during AAA formation (50). The possible mechanisms linking the lipoprotein modifications that we observed in AAA and aortic aneurysm pathology will be discussed below.

Smoke is a known strong risk factor for AAA development (32). Comparing CEC values in smokers and non-smokers, we found significant differences exclusively in the AAA group. In smokers, ABCG1-CEC and ABCA1-CEC were significantly lower and higher, respectively, compared to non-smokers. Although the very small number of patients included in this analysis does not allow to draw conclusions, these results suggest that smoke might emphasize HDL particles modifications specific of AAA patients. This concept is supported by a large clinical study in which smoke cessation was associated with an increase in serum mature HDL levels after 1 year (51). However, the lack of impact of smoking on LCAT and CETP activity suggests the involvement of a different mechanism. The fact that smoking modulates HDL function exclusively in AAA patients suggests that the genetic background in this group may play an important permissive role. The potential relevance of these findings is underlined by the consideration that smoke and genetic background are the major risk factors for the AAA development (52).

CLC, a marker of overall serum effect on intracellular cholesterol content, correlating to serum atherogenic properties in the context of cardiovascular disease studies, (53) did not differ in AAA and control patients. On one side, the lack of difference in CLC between AAA and control patients is not surprising, as both these groups were selected for having the same cardiovascular risk/disease and the same LDL-C serum levels. However, this finding is relevant because it indicates that LDL alterations and dysfunction are not specific features of AAA patients.

Interestingly, only within the AAA group serum CLC correlated directly with ABCG1-CEC and AD-CEC, mainly dependent on mature HDL, and inversely with ABCA1-CEC and CETP activity. The positive correlation of CLC with mature HDL particles suggests that this HDL subpopulation, besides being dysfunctional in terms of CEC in AAA, might even deliver cholesterol to cells (54). The inverse correlation of CLC with ABCA1-CEC and CETP activity found only in AAA is again consistent with the existence of a link between accelerated HDL metabolism, intracellular cholesterol, and aneurysm formation.

The demonstration of the causal mechanisms underlying the relationship between lipoprotein dysfunction and AAA development is beyond the scope of the present study. Moreover, our data do not provide demonstration that the alterations in HDL metabolism and function found in AAA patients have an impact on arterial tissue inflammation and repair. However, a large body of evidence from the literature is available, indicating that altered HDLs induce modifications in tissue homeostasis due to the failure of CEC. Such modifications include, for example, the switch of macrophages toward the proinflammatory and pro-apoptotic M1 phenotype (55, 56), the control of angiogenesis (57), regulation of smooth muscle cell function, and metalloprotease secretion (58). Indeed, inflammation is considered very important in the arterial wall modifications leading to aneurysmatic dilatation, and an altered polarization of arterial macrophages in AAA has been reported (8). In addition, MMP-9 increased expression and elastic fiber destruction have been reported (50, 59). Dysfunctional HDLs are known to carry some inflammatory proteins able to activate processes involved in AAA development. One of such proteins, serum amyloid A (SAA), correlates with early-stage AAA abdominal aortic diameter (60). Indeed, SAA is involved in the regulation of leukocyte chemotaxis, inflammatory cytokine secretion, and MMP expression, thus influencing extracellular matrix remodeling (61, 62). These processes are implicated in AAA formation and progression. Consistently, a robust and inverse relationship between SAA HDL content and ABCG1-CEC, the specific pathway that significantly dropped in our AAA patients, has been documented (49). Interestingly, in our results as in a previous study (25), serum CRP levels did not differ between AAA and control patients, indicating that systemic inflammation is not a major player in AAA formation.

Our hypothesis is that in the context of a genetic background conditioning the abnormal HDL remodeling and smoke sensitivity, with the contribution of factors such as smoke and local shear stress, chronic disturbances in abdominal aortic wall cholesterol fluxes and uncontrolled inflammation might contribute to arterial structure derangement and dilatation.

Our study has several limitations. One of these is the small number of subjects involved, which was, however, sufficient to detect significant differences between the two groups of patients. Another possible limitation is the slight age difference between the two groups, even though lipid levels and cardiovascular disease, the main source of age-related theoretical bias, were similar. Moreover, age did not correlate with any of the studied parameters of HDL metabolism and function. Finally, our data do not directly demonstrate but only suggest the possible mechanisms linking HDL alterations, tissue inflammation, and AAA development.

## Conclusions

We demonstrated that AAA associates with specific alterations of HDL metabolism and function, known to influence intracellular signaling of arterial wall cells, absent in vasculopathic patients with stenotic atherosclerosis. There is a great need to improve early diagnosis and treatment for AAA, before surgery indication. Although we did not demonstrate a causal relationship between lipoprotein alterations and aortic aneurysm development, our data suggest that future studies aimed at validating HDL-related parameters as diagnostic markers or therapeutic targets are worth performing.

## Data availability statement

The raw data supporting the conclusions of this article will be made available by the authors, without undue reservation.

## Ethics statement

The studies involving human participants were reviewed and approved by Ethical Committee of the Faculty of Medicine in Plzen (approval on 12/04/2014). The patients/participants provided their written informed consent to participate in this study.

## Author contributions

NR, IH, and JM conceived the study. NR and MPA designed the experiments, interpreted the results, and wrote the manuscript. FB, FZ, and VT contributed to the experimental design, data interpretation, and manuscript editing. CM, MPA, FZ, MP, AO, and MT performed experiments included in this manuscript. All authors contributed to the article and approved the submitted version.

## Funding

This work was supported by Charles University Research Fund Progres Q39. The funding source had no involvement in study design, in collection, analysis, and interpretation of data or in the writing of the report and in the decision to submit the article for publication.

## Conflict of interest

The authors declare that the research was conducted in the absence of any commercial or financial relationships that could be construed as a potential conflict of interest.

## Publisher’s note

All claims expressed in this article are solely those of the authors and do not necessarily represent those of their affiliated organizations, or those of the publisher, the editors and the reviewers. Any product that may be evaluated in this article, or claim that may be made by its manufacturer, is not guaranteed or endorsed by the publisher.

## Abbreviations

AAA, abdominal artery aneurysm; LCAT, lecithin-cholesterol acyltransferase; CETP, cholesteryl ester transfer protein; CEC, cholesterol efflux capacity; ABCG1, ATP-binding cassette G1; ABCA1, ATP-binding cassette A1; AD, aqueous diffusion; CLC, cholesterol loading capacity.
