# Supplementary figures and images for: HDL metabolism and functions impacting on cell cholesterol homeostasis are specifically altered in patients with abdominal aortic aneurysm
Source: Front Immunol. 2022 Sep 12;13:935241. doi: 10.3389/fimmu.2022.935241 (PMC9510680; doi:10.3389/fimmu.2022.935241)

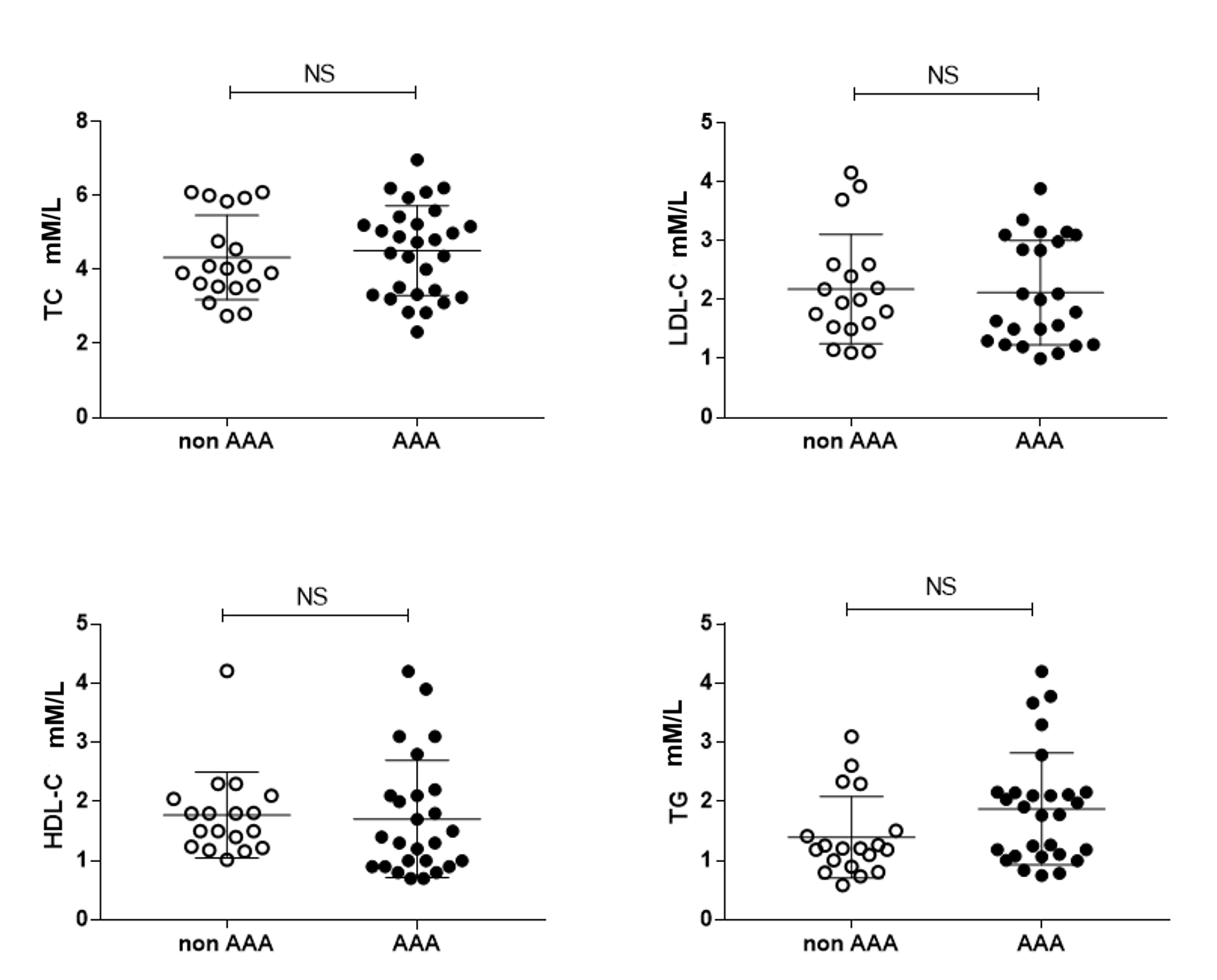

Supplement: Supplementary file 1 [file Image_1.jpeg]
